# Identification and validation of the prognostic value of cyclic GMP-AMP synthase-stimulator of interferon (cGAS-STING) related genes in gastric cancer

**DOI:** 10.1080/21655979.2021.1911557

**Published:** 2021-04-12

**Authors:** Kui-Sheng Yang, Chuan-Qi Xu, Jian Lv

**Affiliations:** Department of General Surgery, People’s Hospital of Jingjiang, Yangzhou University Medical Academy, Jingjiang, China

**Keywords:** Gastric cancer, cGAS-STING pathway, tumor immune, prognostic model

## Abstract

The cyclic GMP-AMP synthase-stimulator of interferon genes (cGAS-STING) pathway play a significant role in the production of inflammatory cytokines and type I interferons. This study aims to develop a cGAS-STING pathway-related genes (CSRs) prediction model to predict prognosis in gastric cancer (GC). In the present study, we used The Cancer Genome Atlas (TCGA), Gene Expression Omnibus databases (GEO), CIBERSORT and Tumor Immune Estimation Resource databases (TIMER). The risk model based on five hub genes (IFNB1, IFNA4, IL6, NFKB2, and TRIM25) was constructed to predict the overall survival (OS) of GC. Further univariate Cox regression (URC) and multivariate Cox regression (MCR) analyses revealed that this risk scoring model was an independent factor. The results were verified by GEO external validation set. Multiple immune pathways were assessed by Gene Set Enrichment Analysis (GSEA). TIMER analysis demonstrated that risk score strongly correlated with Macrophage, B cells and CD8 + T cells infiltration. In addition, through ‘CIBERSORT’ package, the higher levels of infiltration of T cell follicular assistance (P = 0.011), NK cells-activated (P = 0.034), and Dendritic cells resting (P = 0.033) exhibited in high-risk group. Kaplan–Meier (K-M) survival analysis illustrated T cells CD4 memory resting and T cells follicular helper infiltration correlated with overall survival (OS) of GC patients in TCGA and GEO databases. Altogether, the risk score model can be conveniently used to predict prognosis. The immunocyte infiltration analysis provided a novel horizon for monitoring the status of the GC immune microenvironment.

**Abbreviations**:TCGA: The Cancer Genome Atlas databases; GEO: Gene Expression Omnibus databases; GC: Gastric cancer; CSRs: cGAS-STING pathway-related genes; DECSRs: Differential expressed cGAS-STING pathway-related genes; PCSRs: Prognosis related cGAS-STING pathway genes; URC: Univariate Cox regression analyses; MCR: Multivariate Cox regression analyses GSEA: Gene set enrichment analysis; TIIC: Tumor-infiltrating immune cell.

## Introduction

Gastric cancer (GC) is a deadly disease ranking the third leading cause of cancer-related death [1,2]. Most GC patients are already in the middle to late stage when diagnosed due to the occult onset and difficult to early diagnosis, leading to 5-year survival outcome is lower than 25% [3,4]. Despite advances in various therapeutic strategies the survival of remains poor. Hence, it is urgently needed to explore new credible prognostic-stratification tool which could be applied to clinical risk assessment. It would help them in identifying GC patients that are at higher risk for relapse and that might be suitable for closer follow-up.

The cGAS-STING DNA sensing pathway has emerged as a key component of the innate immune response. The STING is in these tumor immune interactions and has pleiotropic effects on tumors [5]. The enzyme cGAS is a universal innate sensor for double-stranded DNA (dsDNA). Upon binding cytosolic dsDNA, cGAS catalyses the synthesis of cyclic GMP-AMP (cGAMP 2ʹ3ʹ), which in turn engages STING to triggers a series of cellular signaling events that consequently lead to the production of type I interferons (IFNs) and inflammatory mediators [1,6,7]. Recently, studies revealed that the cGAS-STING pathway is closely related to tumor immunity. For instance, cGAS-STING pathway activation with antigen-presenting cells leads to production of Tap2 and MHC-I, which may enhance the tumor immune surveillance [8]. Stimulation of the cGAS-STING signaling pathway increased type I IFNs and tumor-infiltrating lymphocytes (TILs) levels to trigger an immunogenic response [9]. cGAS-STING-mediated type I interferon signaling augmented stem cell-like CD8 T cell differentiation and promotes antitumor T cell therapy. These findings suggest that cGAS-STING pathway genes are potential therapeutic target and may be associated with immune infiltration in patients with GC. However, the prognostic value of CSRs is currently lacking in GC.

In our study, we aimed to develop a CSRs prediction model to predict prognosis in GC. Subsequently, survival analysis, ROC curve, Nomogram, univariate and multivariate Cox regression analyses revealed that this risk scoring model was an independent factor. The results were verified by GEO external validation set. Our study may help monitoring the status of the GC immune microenvironment and provide potential targets for the immunotherapy.

## Materials and methods

### Data processing

The work-flow of this study is illustrated in Figure S1A. 375 GC samples and 32 normal tissues gene expression (RNA-seq) were retrieved from TCGA (https://www.cancer.gov/) [10].In addition, the validation cohort microarray (GSE84437) were obtained from the GEO database(https://www.ncbi.nlm.nih.gov/geo/) [11]. The clinical information is shown in the flow Table 1.

**Table 1.** The clinical characteristics of the patients

Items	Databases		
	TCGA		GEO
age	>65	204	150
	≤65	163	283
	Unknow	3	-
Gender	Female	133	137
	Man	237	296
M	M0	327	-
	M1	25	-
	Mx	18	-
N	N0	108	80
	N1	96	188
	N2	74	132
	N3	74	33
	Nx	16	-
Stage	I	49	11
	II	111	38
	III	149	92
	IV	38	292
Treatment	Pharmaceutical Therapy	185	-
	Radiation Therapy	185	-

### CSRs extract and DIfferential expressed genes (DEGs) analysis

117 CSRs were identified via the ‘cGAS-STING’ gene set from the PathCards database (https://pathcards.genecards.org/). The differential expressed cGAS-STING pathway-related genes (DECSRs) were screened with the cutoff: |logFoldChange (logFC)| > 0.5 and adjusted P value < 0.05. ‘ggplot2’ and ‘pheatmap’ packages generated Volcano plots and heat maps, respectively.

### Function enrichment analysis of DECSRs

We also performed Gene Ontology (GO) functional annotations and Kyoto Encyclopedia of Genes and Genomes (KEGG) pathway enrichment analysis use of ‘clusterProfiler’ package in DECSRs [12]. The diagram was made by the R language tool.

### Establishment and validation of the model

We next adopted UCR was used to estimate overall survival (OS) of CSRs. Afterward, MCR was carried out to construct our prognostic risk model and correlation coefficient. Finally, the risk score was calculated as follows: the risk score = ∑i =  1 n(Expi*Coei).

### Independence of the risk model

URC and MRC analysis were conducted to identify independent risk factors for survival. The ‘SurvivalROC’ of R package was constructed to assess the survival differences between groups. The nomogram was established to assess the survival probability for GC patients at 1, 2, and 3 years.

### GSEA

GSEA was also used to analyze the differences pathways between the high-risk and low-risk groups. The C2.cp.kegg.v7.0.symbols.gmt dataset was obtained from the Molecular Signatures Database (MsigDB). NOM P-value <0.05, |NES| >1 and FDR q < 0.25 were considered statistically significant.

### Immune infiltration analysis

TIMER database calculated the infiltration level of 6 immune cells in tumor samples from TCGA database, including B cell, T cell CD4+, T cell CD8+, Neutrophil, Macrophage, and Myeloid dendritic cell [13]. It was used to explore the association of the risk scores and the abundance of six immune cells types (https://cistrome.shinyapps.io/timer/).

### Analysis of immune infiltration in high and low-risk groups

The CIBERSORT applied to estimate the proportions of tumor-infiltrating immune with a deconvolution algorithm. Violin plots were utilized to visualize the distribution of the differences in 22 types of infiltrating immune cells [14].

### Statistical analysis

R software (version 4.0.3) and SPSS software (version 24.0) were used to complete all the statistic work. The survival curve was constructed using survival package via the K-M method. The R package ‘survivalROC’ was employed to analyze prediction efficiency in two groups. P value < 0.05 was considered the cutoff value for significance.

## RESULTS

This study aimed to develop a CSRs prediction model to predict prognosis in GC. Then, UCR and UCR analyses revealed that this risk scoring model was an independent factor. The results were verified by GEO external validation set. Finally, TIMER analysis demonstrated that risk score strongly correlated with immune infiltration. Our study may help monitoring the status of the GC immune microenvironment and provide potential targets for the immunotherapy.

### Various genes differentially expressed with GC progression

**Figure 1.**
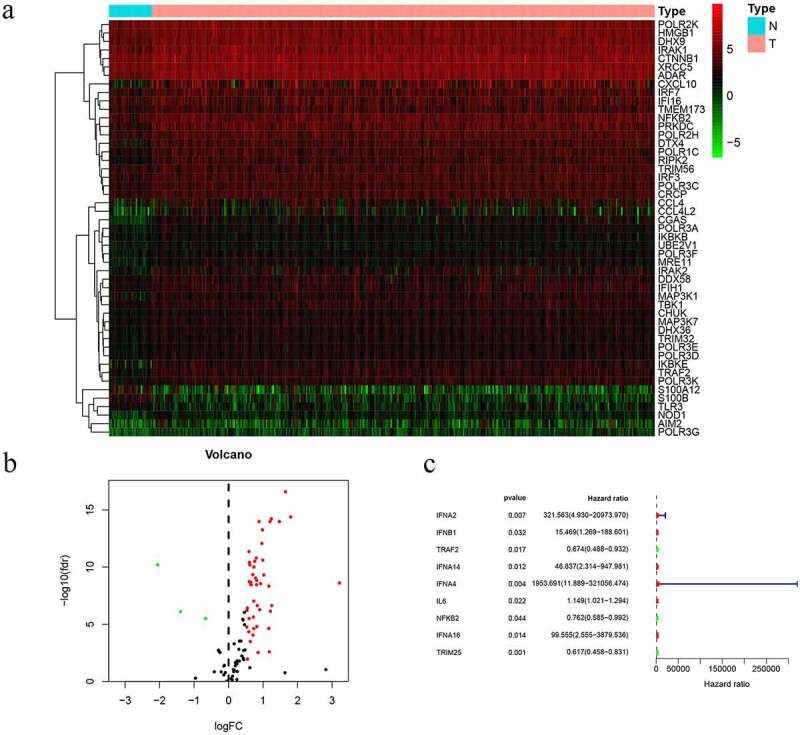
Prognostic differentially expressed CSRs. a. Heatmap of differentially expressed CSRs. b. Volcano plots of differentially expressed CSRs. c. UCR analysis for the CSRs identification in the TCGA patient cohort

In the study, we collected 32 normal tissues and 375 tumors of GC from TCGA database. The 49 differentially expressed CSRs, 46 genes were upregulated, while 3 genes were downregulated and drawing differential heatmap and volcano plot (Figure 1a and b). UCR analysis determined that nine prognosis-related cGAS-STING pathway genes (PCSRs) were independently associated with GC patient OS (Figure 1c).

### Gradually upregulated/downregulated genes involved in multiple immune-related functions and pathways

**Figure 2.**
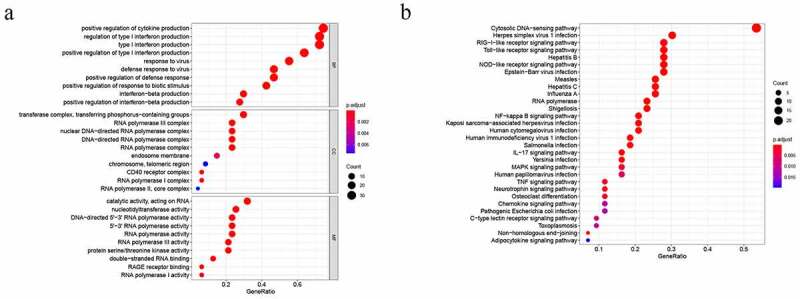
Biological function analysis of differentially expressed CSRs: GO analysis (a) and KEGG pathways analysis (b)

To better understand the biological significance, we conducted enrichment analysis of the 49 differentially expressed CSRs. In molecular function (MF), the main functions of these genes were catalytic activity, acting on RNA, nucleotidyltransferase activity, RNA polymerase activity, and 5ʹ−3ʹ RNA polymerase activity. Cellular component (CC) mainly involved transferase complex, transferring phosphorus−containing groups, RNA polymerase III complex, nuclear DNA−directed RNA polymerase complex, and DNA−directed RNA polymerase complex. In biological processes (BP), the functions of these genes were mainly involved in positive regulation of cytokine production, regulation of type I interferon production, type I interferon production, positive regulation of type I interferon production, and response to virus (Figure 2a). Besides, KEGG pathway annotation showed that these differentially expressed CSRs were significantly enriched in Cytosolic DNA−sensing pathway, RNA polymerase, RIG−I− like receptor signaling pathway, Toll−like receptor signaling pathway and NF−kappa B signaling pathway (Figure 2b).

### Establishment and validation of the prognostic risk model

**Figure 3.**
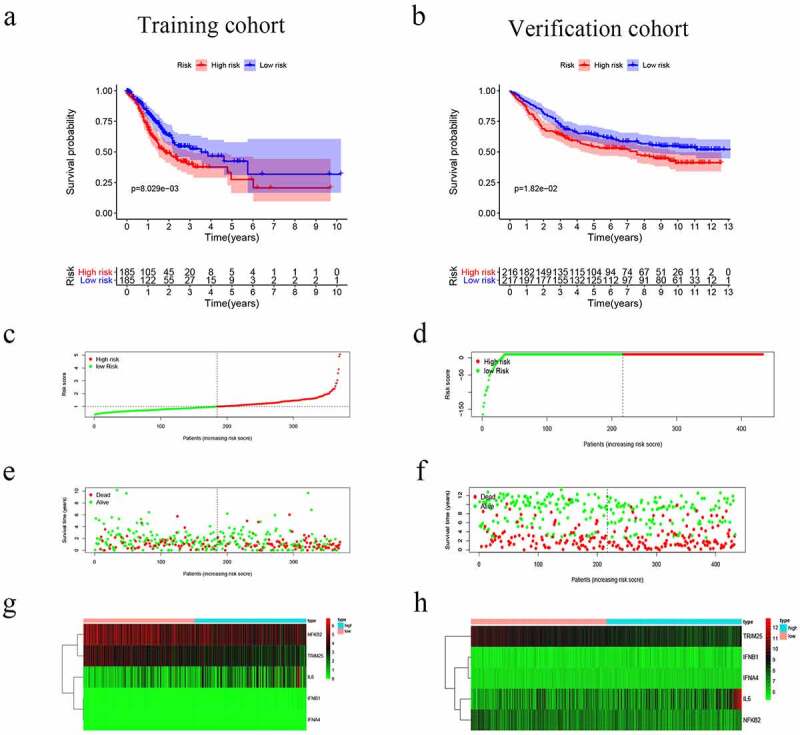
Risk score, reflecting overall survival, based on the CSRs signature comprising five genes, in the training and validation cohorts. (a–b) Kaplan-Meier curve for OS of patients with high- and low risk scores in the training and validation cohorts. (c–d) Risk-score distribution in the training and validation cohorts. (e–f) The survival status plot associated with risk score in the training and validation cohorts. (g–h) Heatmap of the expression of the five CSRs in the high- and low-risk groups and the training and validation cohorts

The nine genes were further analyzed by MCR analysis, and finally, five genes (IFNB1, IFNA4,IL6,NFKB2 and TRIM25) related to the prognosis of GC were obtained. The coefficients of each gene are shown in Table 2. Risk scores = (2.180× IFNB1 Exp) + (4.871× IFNA4 Exp) + (0.135× IL6 Exp) + (−0.366× NFKB2 Exp) + (−0.466× TRIM25 Exp). Patients were divided into high-risk (n = 185) and low-risk (n = 185) groups according to the media risk score. Low-risk patients had a significantly longer OS compared with the patients in high-risk group (Figure 3a). The riskScore plot and survival time and status plot are shown in Figure 3c and 3e, respectively. In addition, to better know the expression level of the five genes in the training set are plotted in Figure 3g. All these evidences were displayed in the GEO validation data set and showed this five genes model was practicable in other independent datasets (Figure 3b, d, f, and h).

**Table 2.** The coefficients of each gene

id	coef	HR	HR.95 L	HR.95 H	pvalue
IFNB1	2.180261	8.848614	0.556624	140.6657	0.122384
IFNA4	4.871504	130.517	0.371338	45,873.81	0.103366
IL6	0.135794	1.145446	1.015096	1.292535	0.027592
NFKB2	−0.36677	0.692966	0.526914	0.911348	0.008687
TRIM25	−0.46663	0.627114	0.462248	0.850782	0.002715

### Independent prognostic value of the risk model

**Figure 4.**
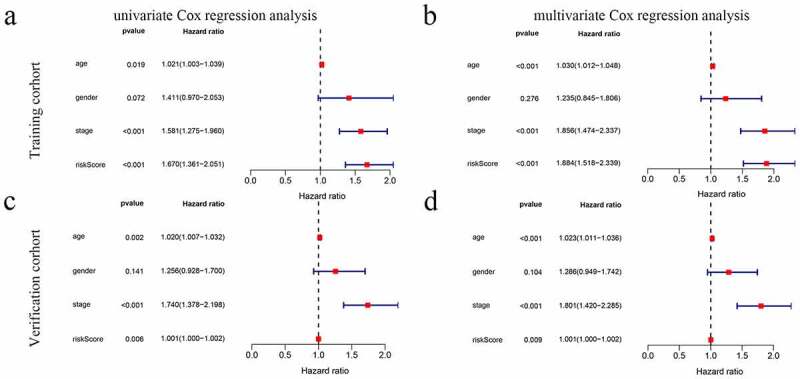
The model was combined with a regression analysis of clinical indicators. (a-b) Assessment of the contribution of each factor to GC survival by UCR and MCR analysis in training cohort. (c, d) Assessment of the contribution of each factor to GC survival by UCR and MCR analysis in verification cohort

The UCR analysis revealed that patients in the high-score group had significantly shorter survival than patients in the low-score group (HR: 1.670; 95% CI: 1.361 − 2.051; P < 0.001). In addition, the clinical variables included age, stage, and stage were significantly associated with survival. A MCR further showed that the risk score (HR: 1.884; CI: 1.518 − 2.339, P < 0.001) was an independent prognostic indicator (Figure 4a and b).

**Figure 5.**
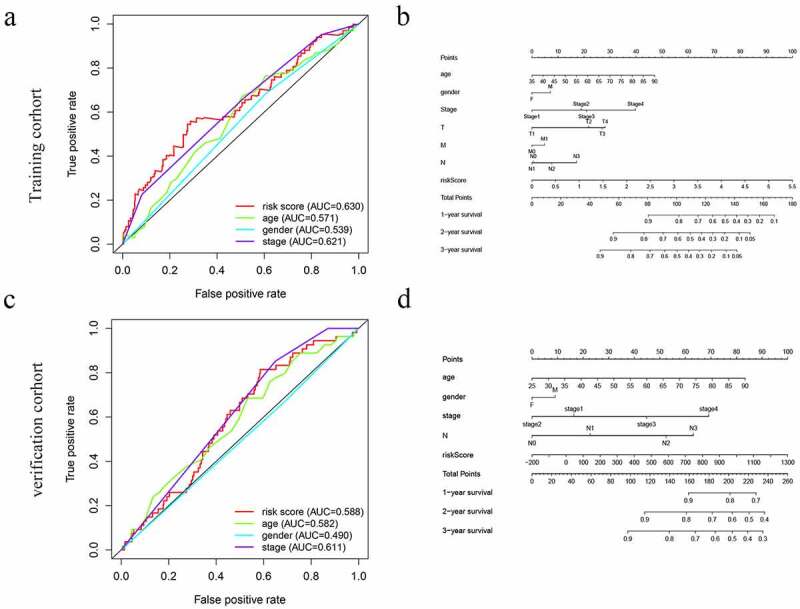
The ROC curves and nomograms for predicting survival rate of GC. (a, c) ROC curves (receiver operating characteristics). (b, d) Nomogram

Prognostic nomogram for the prediction of 1-, 2-, and 3-year overall survival of patients with GC was constructed base on prognostic model and clinical characteristics. (Figure 5b). The total points of each patient provided the estimated 1-, 2-, and 3-year survival times. indicated an ideal fitting and excellent accuracy of the nomogram. These results indicated that the nomogram demonstrated good accuracy prediction of GC patients. Moreover, we also plotted the ROC curves were performed to evaluate the accuracy of the models, with the area under the curve (AUC) scores ranged from 0.539 to 0.630 (Figure 5a).

The efficacy of the prognostic model was validated by another independent cohort GSE84437, we applied the same formula to verification cohort from the GEO cohort. This finding was consistent with the results of the training cohort (Figure 4c and d) (Figure 5c and d).

### High risk group involved in multiple immune-related pathways

**Figure 6.**
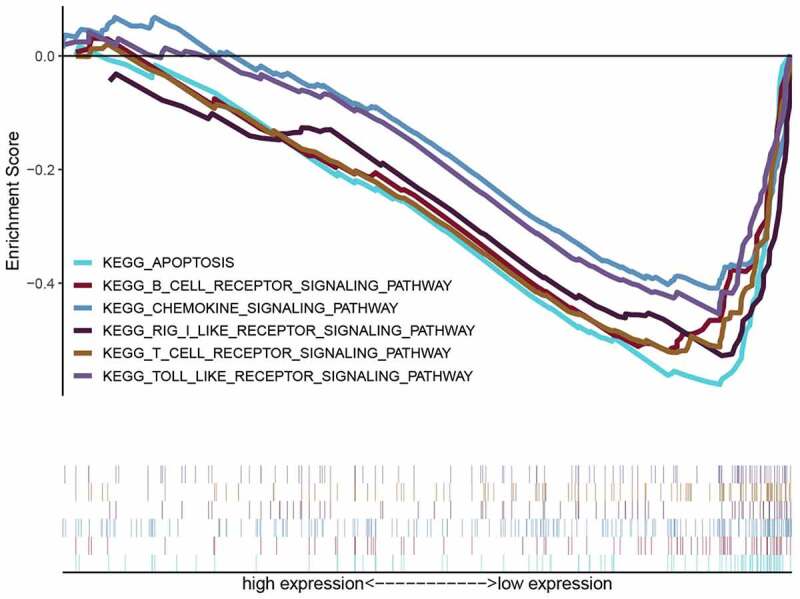
The GSEA of possible pathways of high-risk groups in GC

KEGG-GSEA suggested that high-risk group was markedly negatively related with immune-related biological processes, including The B cell receptor signaling pathway, Chemokine signaling pathway, RIG-I-like receptor signaling pathway, T cell receptor signaling pathway and Toll-like receptor signaling pathway (Figure 6 and Table 3).

**Table 3.** cGAS-STING pathway related gene sets that associated with high-risk group

NAME	NES	NOM p-val	FDR q-val	FWER p-val
KEGG_T_CELL_RECEPTOR_SIGNALING_PATHWAY	−1.904	0.006	0.069	0.163
KEGG_B_CELL_RECEPTOR_SIGNALING_PATHWAY	−1.760	0.031	0.113	0.404
KEGG_TOLL_LIKE_RECEPTOR_SIGNALING_PATHWAY	−1.736	0.025	0.130	0.458
KEGG_CHEMOKINE_SIGNALING_PATHWAY	−1.566	0.048	0.184	0.743
KEGG_APOPTOSIS	−2.238	0.000	0.003	0.002
KEGG_RIG_I_LIKE_RECEPTOR_SIGNALING_PATHWAY	−2.194	0.000	0.003	0.004

ES, enrichment score; NES, normalized enrichment score; NOM, nominal; FDR, false discovery rate.

### Risk scores association with immune infiltration

**Figure 7.**
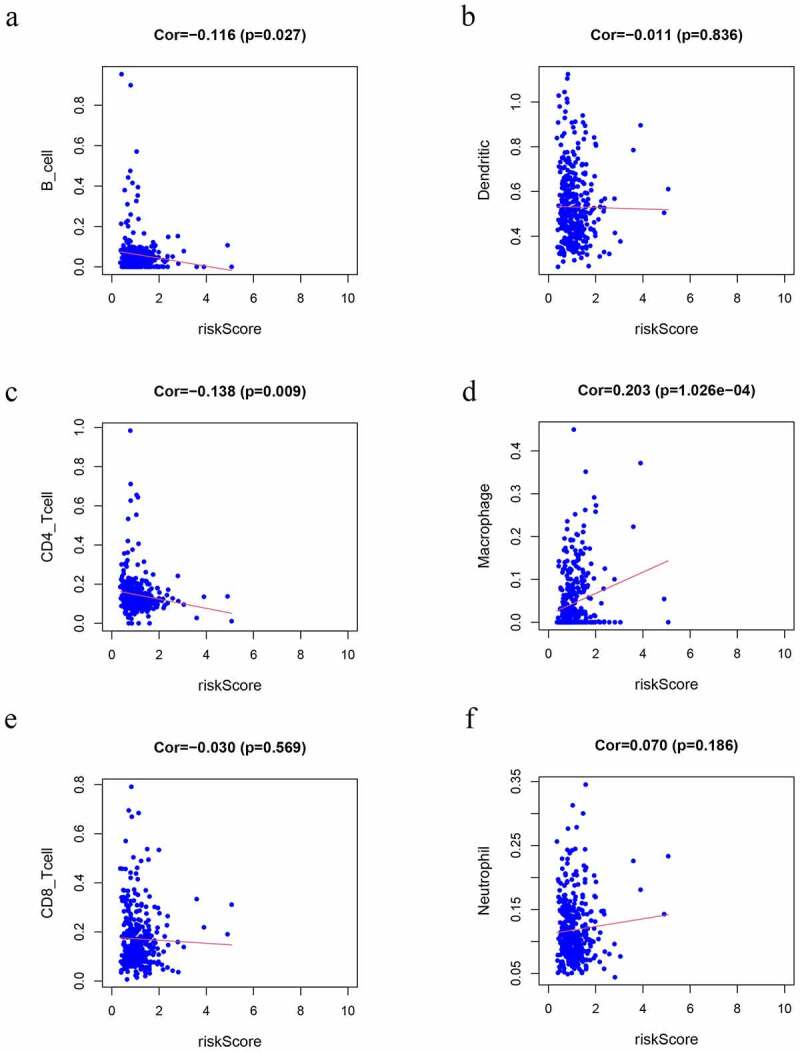
Correlation analysis of the risk score model and immune cell infiltration. (a) B cells, (b) DCs, (c) CD4 + T cells, (d) macrophages, (e) CD8 + T cells, (f) Neutrophils

We observed that risk scores had a positive relationship with the infiltrating levels of the macrophages (r = 0.203 P = 1.026e-4) (Figure 7d). In contrast, CD4 + T cells (r = −0.138 P = 0.009) (Figure 7c) and B cells (r = −0.116 P = 0.027) (Figure 7a) had a negative relationship with risk scores (P < 0.05), which may provide a novel horizon for investigating the GC immune infiltration.

### Analysis of risk groups immune microenvironment

**Figure 8.**
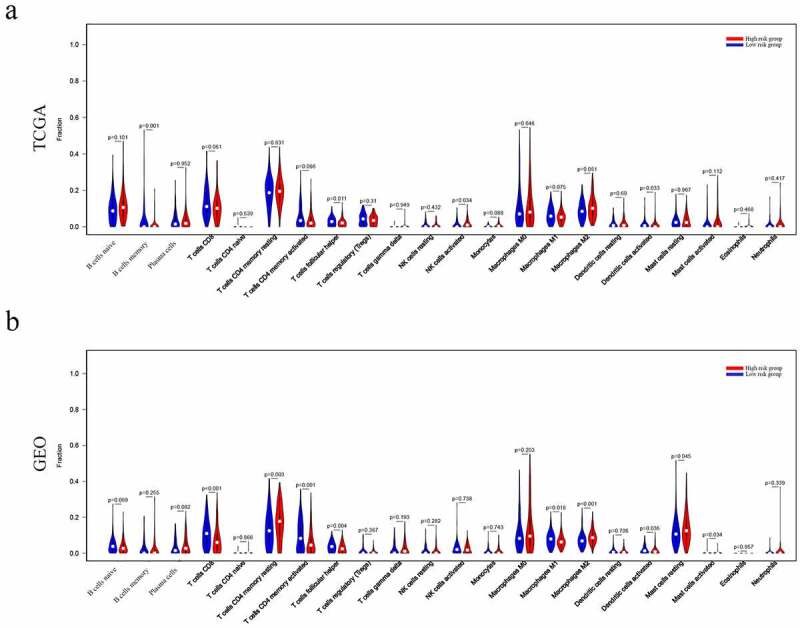
Immune landscape of patients from High- and Low-Riskgroups. Differences of 22 subtypes of immune cells between two groups of TCGA dataset(a), and GEO database (b)

The relationship between risk groups and the tumor immune microenvironment was further analyzed. As a result, the results indicated that many immune cell types were significantly altered among groups, including B cells memory, T cell follicular assistance, NK cells activated, and Dendritic cells resting (P = 0.001, 0.011 0.034, and 0.033, respectively) in TCGA dataset (Figure 8a). Similarly, T cells CD8, T cells CD4 memory resting, T cells CD4 memory activated, T cells follicular helper, Macrophages M1, Macrophages M2, Dendritic cells activated, Mast cells activated and Mast cells resting (P = 0.0001, 0.003, 0.001, 0.004, 0.018, 0.0001, 0.036, and 0.034, respectively) (P = 0.045) were significantly altered among groups in GEO dataset (Figure 8b).

### Identification of prognostic subtypes of tumor-infiltrating immune cells (TIICs) in GC

**Figure 9.**
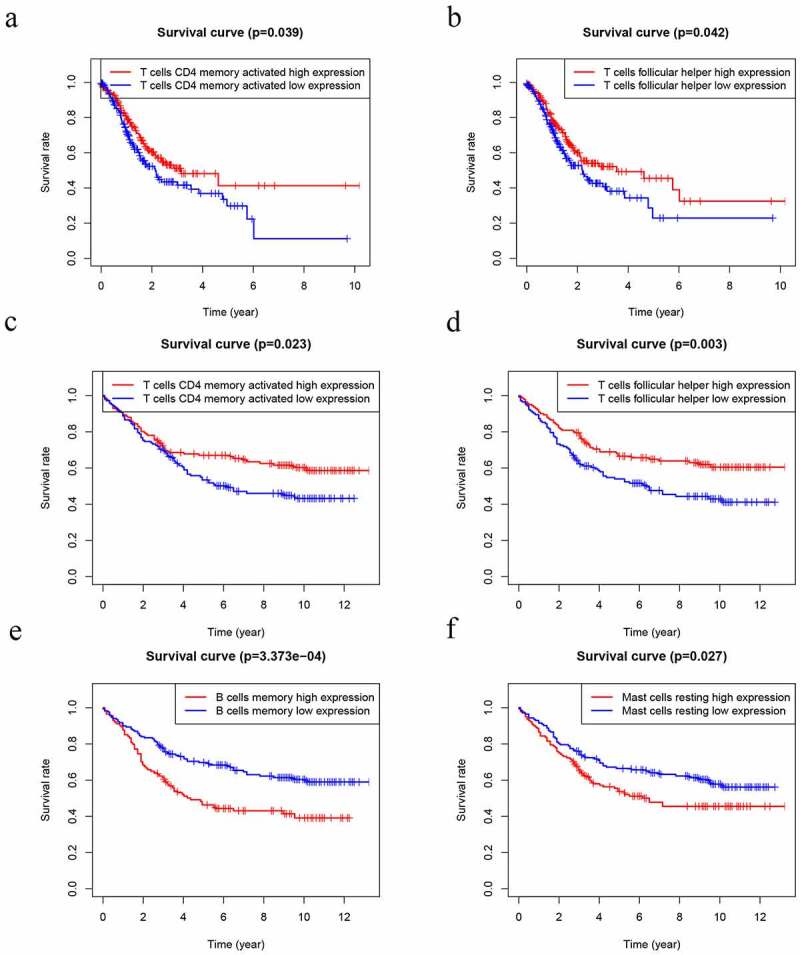
Kaplan–Meier analysis of 22 kinds of immune cells. (a-b) The levels of T cells CD4 memory activated (a) and T cells follicular helper (b) were significantly associated with OS in patients with GC in TCGA database. (d-f) The levels of T cells CD4 memory activated (c), T cells follicular helper (d), B cells memory (e) and Mast cells resting (f) were significantly associated with OS in patients with GC in GEO database

Several studies have already demonstrated that TIICs correlates with prognosis in several malignancies [15]. Thus, we performed K-M analysis that tried to investigate the prognostic subtypes of TIICs. The outcome uncovered that T cells follicular helper (P = 0.039) and T cells CD4 memory activated (P = 0.042) were positively associated with worse OS of GC patients in TCGA datasets (Figure 9a and b). In GEO datasets, T cells follicular helper (P = 0.003) and T cells CD4 memory activated (P = 0.023) were positively associated with worse OS (Figure 9c and d). However, B cells memory (P = 3.373e-4) and Mast cells activated (P = 0.027) were negatively associated with worse OS (Figure 9e and f).

## Discussion

The cGAS-STING pathway has emerged as a potential mechanism to induce inflammation-mediated tumorigenesis. Actually, persistent activation of this pathway and its downstream effectors, such as TBK1, has been connected with chronic inflammation and cancer progression [2, 16]. The development of GC is a multistep process that involves continuous inflammatory damage. Some studies have observed correlations between cGAS-STING pathway members, the tumor microenvironment, and cancer immunotherapy. Recent studies have analyzed that the STING activation promotes natural killer (NK) cells and CTLs responses against tumors [17]. The activation of STING pathway is accompanied by down-expression of several immune inhibitory factors, including PD-L1, IDO, and FOXP3 [18]. Lower expression level of STING, and STING expression levels are positively correlated with prognosis, that is, a higher STING expression level results in a better prognosis in GC patients. Therefore, the expression of CSRs was dysregulated in GC and played a crucial role in progression and prognosis of GC patients.

In our study, UCR and MCR analyses were applied to construct an CSRs risk model. Subsequent K-M analysis revealed that patients with higher risk scores exhibited lower OS. We further validated the sensitivity and accuracy of the model in the GEO database. Furthermore, our GSEA data analysis revealed that the immune pathways were significantly enriched in the high-risk group, such as The B cell receptor signaling pathway, Chemokine signaling pathway, T cell receptor signaling pathway and Toll-like receptor signaling pathway.

IFNB1 and IFNA4 belong to the type I IFNs. IFN-β1 direct anti-angiogenic, and anti-tumor on the one hand, and stimulate immune production on the other hand [19]. Recently, a vesicular stomatitis virus expressing IFN-β1 was able to create a ‘comfortable’ tumor microenvironment for immune checkpoint inhibition [20]. IL6 is a pro-inflammatory cytokine, which acts in the initiation of innate immune responses [21]. Moreover, stromal IL6 promotes cancer immune-evasive microenvironment through metabolic reprogramming [22]. NFKB2 gene is part of the NFKB pathway family genes, which is an important regulator in immune reactivity in various types of cancer including GC [23]. Notably, it has been shown that NFKB directly regulates PD-L1 transcription by binding to the PD-L1 promoter [24]. One of the TRIM family members, TRIM25, participates in the regulation of biological processes, including tumor cell proliferation, invasion and migration [25,26]. In addition, decreased TRIM25 expression in tumor tissues were positively correlated with poor prognosis of GC patients [27].

Another important finding in the present study that there is a significant correlation between the risk scores and the macrophages, CD4 + T cells and B cells infiltration. Moreover, we identified differential expression of three types of immune cells between High- and Low-Risk groups. In addition, two types of TIICs are associated with OS of GC patients. Recent studies have shown that the higher CD4 + T cell density and CD4/CD8 ratio were associated with worse OS in tumor, including ductal carcinoma in situ, glioblastoma, and GC [28–30]. Tumor-infiltrating B cells play a critical role in regulating the anti-tumor immune response in melanoma, and the absence of B cells is associated with a poor response to immune checkpoint inhibitors (ICIs) [31,32]. CD8 + T cells constitute an important part of the immune response to tumors and play a critical role in killing tumor cells [33]. Macrophages could display antitumour M1 and protumour M2 phenotypes, and high density of M1 macrophages was associated with better overall survival in GC [34]. This implies that cGAS-STING related genes prognostic model may act as potential prognostic indicators, as well as reflect the immune status. Collectively, this newly identified cGAS-STING-related genes risk score signature based on the combination of five genes could significantly predict the prognostic risk and might provide insight into immunotherapy in GC.

## Conclusion

In summary, we developed a cGAS-STING pathway-related prognostic index of GC and the risk score model can be conveniently used to predict prognosis, and we found that the underlying molecular mechanisms may affect immune-related biological processes and TIICs, which may provide novel insights into the relationship between GC and tumor immune infiltration. However, these results need to be further validated in future studies.

## Supplementary Material

Supplemental Material

## Data Availability

The datasets analyzed was acquired from The Cancer Genome Atlas (TCGA) database (https://portal.gdc.cancer.gov/) and GEO database (https://www.ncbi.nlm.nih.gov/geo/).

## Highlights

(1) A cGAS-STING pathway-related prognostic index to predict the prognosis of GC was constructed.

(2) The prognostic index is an independent index for GC prognosis.

(3) The prognostic index may affect immune-related biological processes and TIICs.
