# Supplementary figures and images for: Identification and validation of the prognostic value of cyclic GMP-AMP synthase-stimulator of interferon (cGAS-STING) related genes in gastric cancer
Source: Bioengineered. 2021 Apr 12;12(1):1238–50. doi: 10.1080/21655979.2021.1911557 (PMC8291813; doi:10.1080/21655979.2021.1911557)

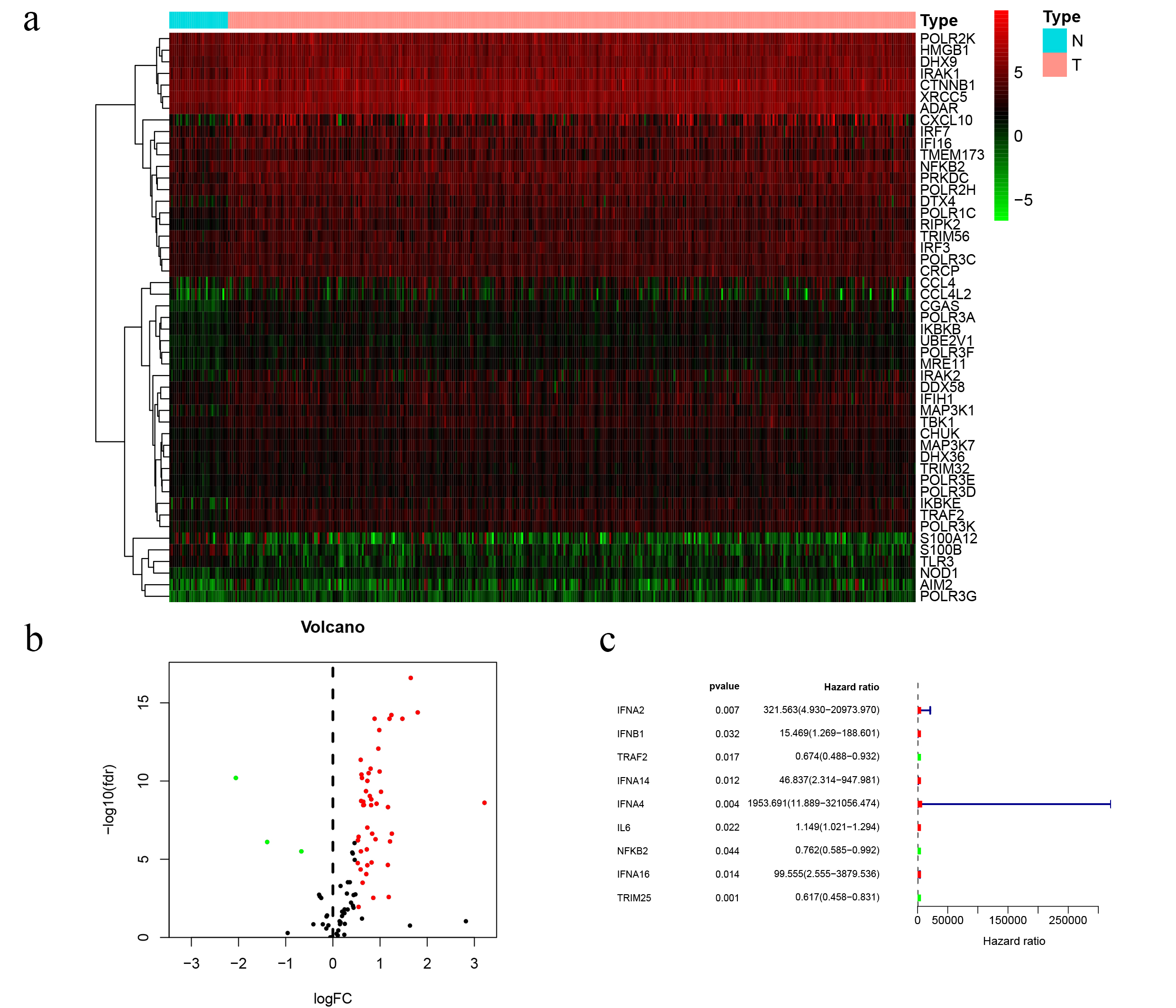

Supplement: Supplemental Material [file KBIE_A_1911557_SM8267.jpg]
